# Effect of Vitamin D on Peripheral Blood Mononuclear Cells from Patients with Psoriasis Vulgaris and Psoriatic Arthritis

**DOI:** 10.1371/journal.pone.0153094

**Published:** 2016-04-06

**Authors:** Susana Cubillos, Nadine Krieg, Johannes Norgauer

**Affiliations:** Department of Dermatology, Jena University Hospital, Jena, Thüringen, Germany; Faculté de médecine de Nantes, FRANCE

## Abstract

**Background:**

Psoriasis, a chronic skin disease with or without joint inflammation, has increased circulating proinflammatory cytokine levels. Vitamin D is involved in calcium homeostasis, bone formation, osteoclastogenesis and osteoclast activity, as well as regulation of immune response. We aimed to study osteoclast differentiation and cytokine secretion of peripheral blood mononuclear cells (PBMCs) from patients with psoriasis vulgaris and psoriatic arthritis, in response to 1,25(OH)_2_D_3_.

**Methods:**

Serum levels of bone turnover markers were measured by ELISA in patients with psoriasis vulgaris and psoriatic arthritis, and healthy controls. PBMCs were isolated and cultured with or without RANKL/M-CSF and 1,25(OH)_2_D_3_. Osteoclast differentiation and cytokine secretion were assessed.

**Results:**

Psoriatic arthritis patients had lower osteocalcin, as well as higher C-telopeptide of type I collagen and cathepsin K serum levels compared with psoriasis vulgaris patients and controls. RANKL/M-CSF-stimulated PBMCs from psoriatic arthritis patients produced higher proinflammatory cytokine levels and had a differential secretion profile in response to 1,25(OH)_2_D_3_, compared with psoriasis vulgaris and control PBMCs.

**Conclusions:**

Our data confirmed altered bone turnover in psoriatic arthritis patients, and demonstrated increased osteoclastogenic potential and proinflammatory cytokine secretion capacity of these PBMCs compared with psoriasis vulgaris and controls. 1,25(OH)_2_D_3_ abrogated these effects.

## Introduction

Psoriasis is a chronic inflammatory skin disease with or without joint inflammation. Phototherapy and topical application of vitamin D analogs are widely used in the treatment of psoriasis vulgaris. Topical vitamin D regulates serum calcium levels and phototherapy alters systemic levels of vitamin D, a crucial factor in the regulation of extracellular calcium homeostasis and bone metabolism [1,2]. Osteoclasts resorb mineralized bone and osteoblasts are responsible for new bone formation. Osteoclasts are multinucleated cells derived from the monocyte/macrophage lineage [3]. Osteoclast differentiation is supported by osteoblasts through cell-to-cell interactions and two major cytokines—receptor activator of NF-ϰB ligand (RANKL) and macrophage-colony stimulating factor (M-CSF) [4–6]. Activation of the receptors RANK and c-Fms on osteoclast precursors by these ligands induces calcium signalling pathways linked to activation of the nuclear factor of activated T cells cytoplasmic 1 (NFATc1) [7,8], which regulates the expression of osteoclast-specific markers such as the type I collagen degrading cathepsin K (CTSK), the osteopontin dephosphorylating tartrate-resistant acid phosphatase (TRAP) and calcitonin receptor [9–11]. This receptor binds to calcitonin (CT), a hormone produced primarily by thyroid C-cells in response to elevated serum calcium levels [10]. CT reduces blood calcium, through inhibition of bone resorption [12] and regulation of 1,25(OH)_2_D_3_ production in the kidney [13]. In addition to the hormonal control of calcium homeostasis, the vitamin D active form 1,25(OH)_2_D_3_ functions on cellular growth, proliferation and differentiation. Locally produced 1,25(OH)_2_D_3_ by osteoblasts is involved in the regulation of osteoclastogenesis and osteoclast activity [14] increasing the expression of RANKL as well as decreasing the expression of its antagonist osteoprotegerin in osteoblasts [15]. Cells of the monocyte/macrophage lineage hydroxylate 25(OH)D_3_ into 1,25(OH)_2_D_3_ [16,17]. In particular, PBMCs-derived osteoclasts also respond to vitamin D through vitamin D receptor with increased NFATc1 expression [18].

Bone and immune cells share bone marrow progenitors and are affected by the same cytokines and metabolites such as 1,25(OH)_2_D_3._ In fact, 1,25(OH)_2_D_3_ inhibits the expression of cytokines such as IL-1, IL-2, IL-6, IL-12, IL-23, interferon γ (IFN-γ), tumour necrosis factor α (TNF-α) and chemokines such as IL-8 and chemokine (C-C motif) ligand 5 (CCL5 or RANTES) by monocytes, T and B cells [19–22]. Conversely, 1,25(OH)_2_D_3_ increases production of IL-10 of activated T and B cells, and interferon β in osteoclast precursors [23,24]. Besides high levels of circulating proinflammatory cytokines [25,26], patients with psoriatic arthritis have higher circulating bone and cartilage degradation products [27,28] and higher number of osteoclast precursors than healthy individuals [29]. Cytokine effects on the osteoclast differentiation and activity are well studied, but knowledge about cytokine secretion pattern of PBMCs derived from patients with psoriasis vulgaris and psoriatic arthritis and their capacity to differentiate into mature osteoclasts is limited. Therefore we aimed to study the osteoclast differentiation and cytokine secretion capacity of PBMCs from psoriasis vulgaris and psoriatic arthritis patients in response to 1,25(OH)_2_D_3_. We found increased osteoclastogenic potential and proinflammatory cytokine secretion capacity of PBMCs from patients with psoriatic arthritis compared with psoriasis vulgaris and controls. In addition, 1,25(OH)_2_D_3_ abrogated these effects.

## Materials and Methods

### Subject characteristics

This study, approved by the Ethics Committee at the Medical Faculty of the Friedrich-Schiller University Jena (Project 1940-01/07), was conducted according to the principles of the Declaration of Helsinki. Written consent was obtained from all participants prior to enrolment. Patients were diagnosed based on clinical and pathological findings at the Department of Dermatology of the Jena University Hospital. All patients fulfilled the CASPAR criteria [30]. The presence of joint manifestations was confirmed with power doppler ultrasonography (Esaote, Italy) and Rheumascan Xeralite (Mivenion GmbH, Germany). Twenty one patients with psoriasis vulgaris and fifteen healthy controls (HC) were included. Nine patients with psoriasis vulgaris had no clinical signs of joint inflammation (PsV) and twelve were diagnosed with psoriatic arthritis (PsA). Demographic data are shown in S1 Table. Patients with other types of psoriasis (guttate, inverse, pustular, erythrodermic), other skin diseases, allergy, autoimmune diseases, any topical or systemic treatment, including vitamin D supplementation or phototherapy 5 months before or at the time of recruitment were excluded.

### Serum levels of bone turnover markers

Human calcitonin (CT) and 1,25-dihydroxyvitamin D_3_ [1,25-(OH)_2_D_3_] (Shanghai Sunred Biological Technology Co., Ltd, China), osteocalcin (OCN) (ALPCO Diagnostics, USA), C-telopeptide of type I collagen (CTX-1) and of type II collagen (CTX-2) (CUSABIO Biotech Co., Ltd, China), and cathepsin K (CTSK) (Uscn Life Science Inc, China) ELISA kits were used according to the manufacturer instructions to assess the respective patient and control serum levels. Detection ranges of ELISA kits were: 0.7–200 mmol/L for CT, 0.7–150 ng/ml for 1,25-(OH)_2_D_3_, 0.31–1250 ng/ml for OCN, 25–800 ng/ml for CTX-1, 0.312–20 ng/ml for CTX-2, and 15.6–1000 pg/ml for CTSK.

### Serum calcium levels

Serum calcium levels were determined using the QuantiChrom calcium assay kit (BioAssay Systems, USA). The assay was used according to the manufacturer instructions to assess the respective patient and control serum levels. The linear detection range was 0.08 to 20 mg/dl.

### Isolation and culture conditions of PBMCs

PBMCs were isolated from blood samples by density gradient centrifugation. In 24-well plates, 1 x 10^6^ cells/ml/well (0.5 x 10^6^ cells per cm^2^) containing alpha MEM medium with 10% fetal bovine serum, 100 units/ml penicillin, and 100 μg/ml streptomycin were placed. Cells were cultured with or without 30 ng/ml RANKL and 25 ng/ml M-CSF (Promokine GmbH, Germany), in the presence or absence of 10 nM calcitriol (1,25(OH)_2_D_3,_ Cayman Chemical, USA). The medium was replenished at 4, 8, 11 and 14 days, and after 14 days cells were fixed for tartrate-resistant acid phosphatase (TRAP) staining and 200 μl/well supernatant six wells of each condition were pooled and stored at -20°C until cytokine levels and TRAP enzymatic activity were measured.

### TRAP activity assay

At 14 days in culture, mature osteoclasts were identified as TRAP+ multinucleated cells and counted per microscope field after incubation with TRAP stain (Sigma-Aldrich, USA) at 37°C for 5 to 10 minutes. In addition, TRAP enzymatic activity in PBMCs culture supernatants was measured at 14 days as follows: a standard curve was made by using known concentrations naphtol (Sigma-Aldrich, USA) as substrate and the supernatant from the sample with the highest amount TRAP+ cells. Samples and the standard curve with TRAP stain solution were added to microtiter plates, incubated at 37°C for 10 hours, and absorbance at 540 nm was measured. The amount of dephosphorylated substrate (nmoles) in the samples was calculated by comparing their absorbance with a 4-parameters-logistic substrate standard curve. Data was normalized against total protein and results were expressed as nmoles/mg protein/hour.

### Cytokine levels in PBMCs culture supernatants

Levels of TNF-α, IL-1b, IFN-γ, IL-17, IL-23, IL-2, RANTES and IL-10 in 14 days culture supernatants were simultaneously and concurrently assessed with Q-Plex multiplex arrays according to manufacturer instructions. At least five samples from each patient and control were tested. Each cytokine concentration in the samples was calculated by comparing the sample chemiluminescence intensities with the chemiluminescence intensities of the standard curves with the Q-View software (QUANSYS Biosciences, USA).

### Statistical analyses

Statistical analyses were performed using the GraphPad Prism software (GraphPad Software, Inc, USA). Differences between groups were analyzed by Mann-Whitney or t-test in the case of two groups, and further one-way analysis of variance (ANOVA) with Dunnetts’s multiple comparison tests in the case of more than two groups. Additionally of Mann-Whitney for two groups, cytokine profiling grouped data were analyzed by two-way ANOVA with *post hoc* Bonferroni test. Spearman’s non-parametric correlation test was also applied. *P*<0.05 was considered statistically significant.

## Results

### Subject demographics

We did not find significant differences in age between HC, PsV and PsA groups (after 1 way ANOVA analysis with Bonferroni’s multiple comparison test). Furthermore, although we found a significant age effect (*P*<0.05) from females in the PsV group (significant older than control females), we did not observe any significant interaction between age and gender after two way ANOVA with Bonferroni post test analysis (S1 Table).

### Serum bone turnover markers

**Fig 1:**
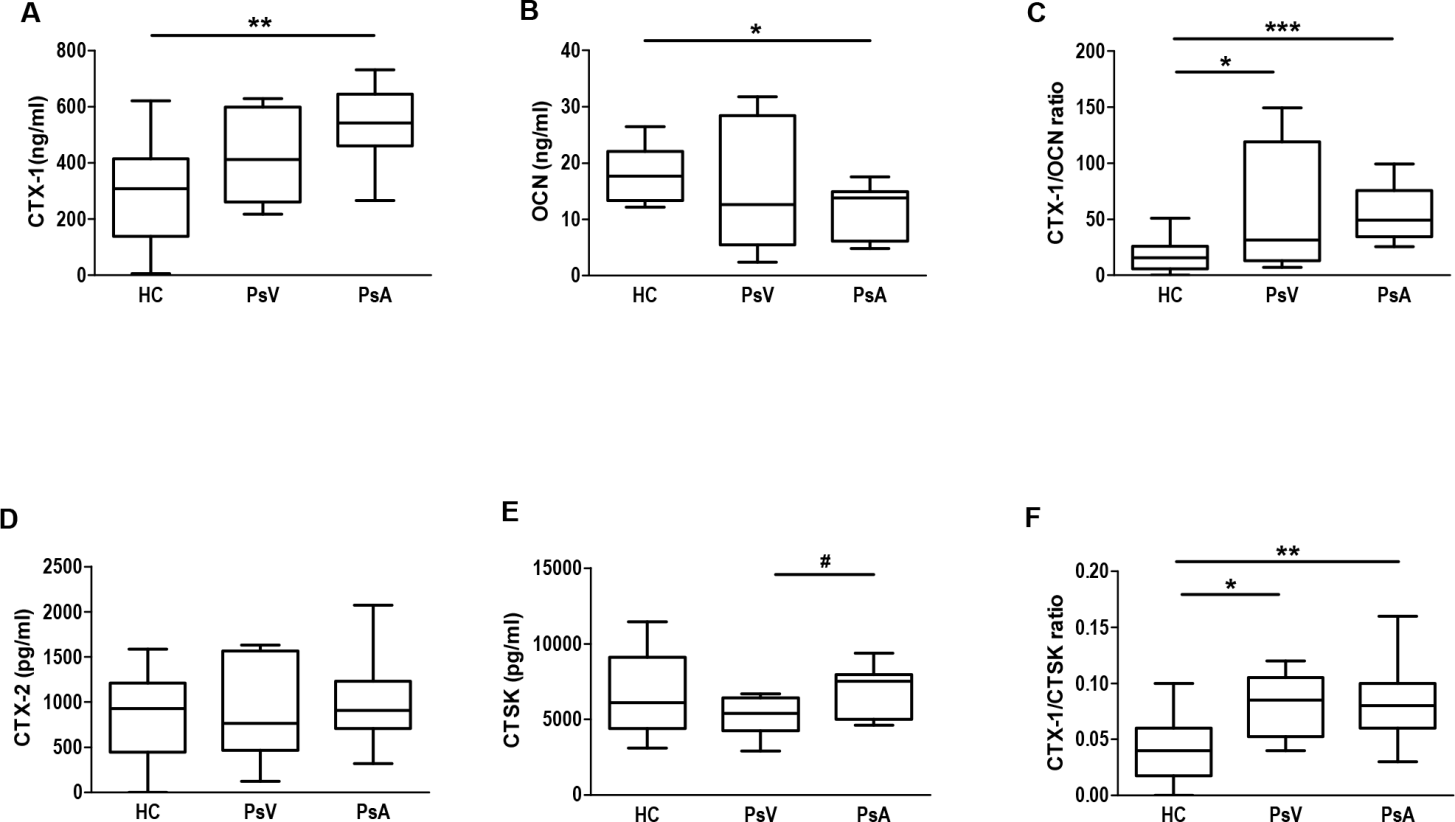
Bone turnover markers in serum from psoriasis patients and controls. Box and whisker plots show serum concentrations of A) CTX-1, B) OCN, C) CTX-1/OCN ratio, D) CTX-2, E) CTSK, and F) CTX-1/CTSK ratio in healthy controls (HC, n = 14), and patients with psoriasis vulgaris (PsV, n = 8) and psoriatic arthritis (PsA, n = 11) measured by ELISA kits. Box and whisker plots represent median with minimum to maximum values. *P<0.05, **P<0.01 and ***P<0.001 indicate statistically significant differences obtained by one-way ANOVA with Dunnetts’s multiple comparison test; and #*P*<0.05 only by t-test.

Product of bone degradation such as CTX-1 and the hormone OCN involved in bone formation are useful markers to assess bone turnover and already reported as bone remodelling markers in patients with PsA [28,31]. Here we assessed serum levels of these markers to confirm bone turnover status in the patient and control samples evaluated. We found no significant differences in CTX-1 and OCN serum levels between patients with PsV and controls; however, higher levels of CTX-1 (*P* = 0.002) and lower levels of OCN (*P* = 0.011) were observed in the serum of patients with PsA compared with controls (Fig 1A and 1B). The CTX-1/OCN ratios were also highly significant in patients with PsA compared with controls (*P* = 0.0004, Fig 1C). In contrast to CTX-1, no significant differences in CTX-2 serum levels were found in any of the analyzed groups (Fig 1D). It is well known that osteoclasts use CTSK to degrade collagen type I, and CTX-1 is a product of this degradation [32]. Here we investigate for the first time CTSK serum levels to assess bone degradation status in patients with psoriasis. In addition to higher CTX-1/OCN ratios in patients with PsA compared with controls, we found also higher levels of CTSK in patients with PsA compared with PsV (*P* = 0.028, Fig 1E). Also, levels of degradation product per enzyme CTSK (CTX-1/CTSK ratios) were highly significant in patients with PsA compared with controls (*P* = 0.002, Fig 1F). Furthermore, we found a significant inverse Spearman’s coefficient correlation between CTSK and CT levels in patients with PsA (r = -0.711, *P* = 0.014) indicating the reciprocal relationship between both parameters (Table 1).

**Table 1:** Spearman coefficient correlation analysis of serum parameters in healthy controls and patients with psoriasis vulgaris and psoriatic arthritis.

Serum	Spearman coeficient (*P* value)		
parameters	HC (n = 14)^a^	PsV (n = 8)^a^	PsA (n = 11)^a^
CTSK vs CT	-0.16 (0.57)	-0.41 (0.32)	**-0.71 (0.01)**
CTSK vs Ca	-0.09 (0.75)	-0.14 (0.74)	-0.09 (0.79)
CTSK vs Vit D	-0.17 (0.55)	-0.48 (0.23)	-0.53 (0.10)
CTSK vs CTX-1	0.42 (0.14)	0.55 (0.16)	-0.22 (0.52)
CTSK vs OCN	0.09 (0.77)	-0.48 (0.23)	0.09 (0.79)
CTSK vs CTX-1/OCN	0.35 (0.22)	0.52 (0.18)	-0.19 (0.57)
CTSK vs CTX-2	0.09 (0.76)	0.50 (0.20)	-0.09 (0.79)
CT vs Ca	-0.35 (0.22)	0.38 (0.35)	-0.10 (0.77)
CT vs Vit D	**0.95 (<0.0001)**	**0.71 (<0.05)**	**0.71 (0.01)**
Ca vs Vit D	-0.21 (0.47)	0.38 (0.35)	0.05 (0.89)
CT vs CTX-1	0.29 (0.31)	-0.43 (0.30)	0.12 (0.72)
CT vs OCN	0.11 (0.70)	0.36 (0.39)	0.11 (0.76)
CT vs CTX-2	-0.12 (0.71)	-0.14 (0.75)	-0.32 (0.34)
Ca vs CTX-1	-0.28 (0.33)	-0.10 (0.82)	0.00 (1.00)
Ca vs OCN	-0.15 (0.62)	0.57 (0.14)	-0.23 (0.50)
Ca vs CTX-2	-0.39 (0.19)	-0.10 (0.82)	0.46 (0.15)
Vit D vs CTX-1	0.32 (0.26)	-0.02 (0.96)	-0.01 (0.98)
Vit D vs OCN	0.01 (0.98)	0.52 (0.18)	0.35 (0.30)
Vit D vs CTX-2	-0.23 (0.45)	-0.71 (0.05)	0.05 (0.89)
CTX-1 vs OCN	-0.17 (0.56)	-0.52 (0.18)	-0.10 (0.77)
CTX-1 vs CTX-2	-0.09 (0.78)	-0.37 (0.37)	-0.04 (0.92)
OCN vs CTX-2	-0.01 (0.99)	-0.13 (0.76)	0.36 (0.29)

Abbreviations: HC, healthy controls; PsV, psoriasis vulgaris; PsA, psoriatic arthritis; CTSK, cathepsin K; CT, calcitonin; Ca, calcium; Vit D, 1,25(OH)_2_D_3_; CTX-1, C-telopeptide of type I collagen; OCN, osteocalcin; CTX-2, C-telopeptide of type II collagen.

^a^n is the number of participants. *P*<0.05.

### Serum levels of 1,25(OH)_2_D_3_, calcium and calcitonin

**Fig 2:**
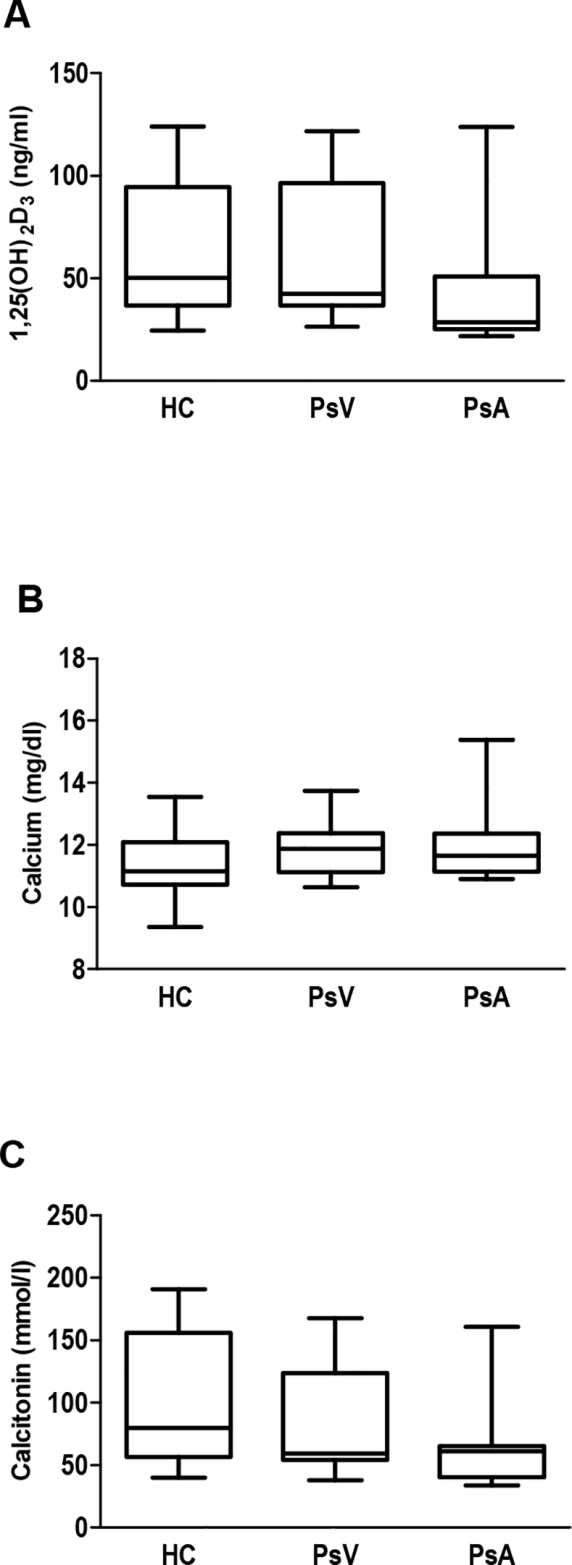
Levels of 1,25(OH)_2_D_3_, calcium and calcitonin in serum from psoriasis patients and controls. Box and whisker plots show serum concentrations of A) 1,25(OH)_2_D_3_, B) calcium and C) calcitonin, in healthy controls (HC, n = 14), and patients with psoriasis vulgaris (PsV, n = 8) and psoriatic arthritis (PsA, n = 11) measured by ELISA kits. Box and whisker plots represent median with minimum to maximum values.

Next, 1,25(OH)_2_D_3_, calcium and CT levels in serum from patients with PsV and PsA, and controls were analysed (Fig 2). No significant differences between these groups were observed as expected. However, we found a significant positive Spearman’s coefficient correlation between CT and 1,25(OH)_2_D_3_ in patients and controls, indicating a direct and very strong relationship between increase or decrease of both variables in controls and a less strong relationship in case of psoriasis patients (HC, r = 0.947, *P*<0.0001; PsV, r = 0.714, *P* = 0.047; PsA, r = 0.711, *P* = 0.014; Table 1).

### Comparison of TRAP enzymatic activity between cultured PBMCs

**Fig 3:**
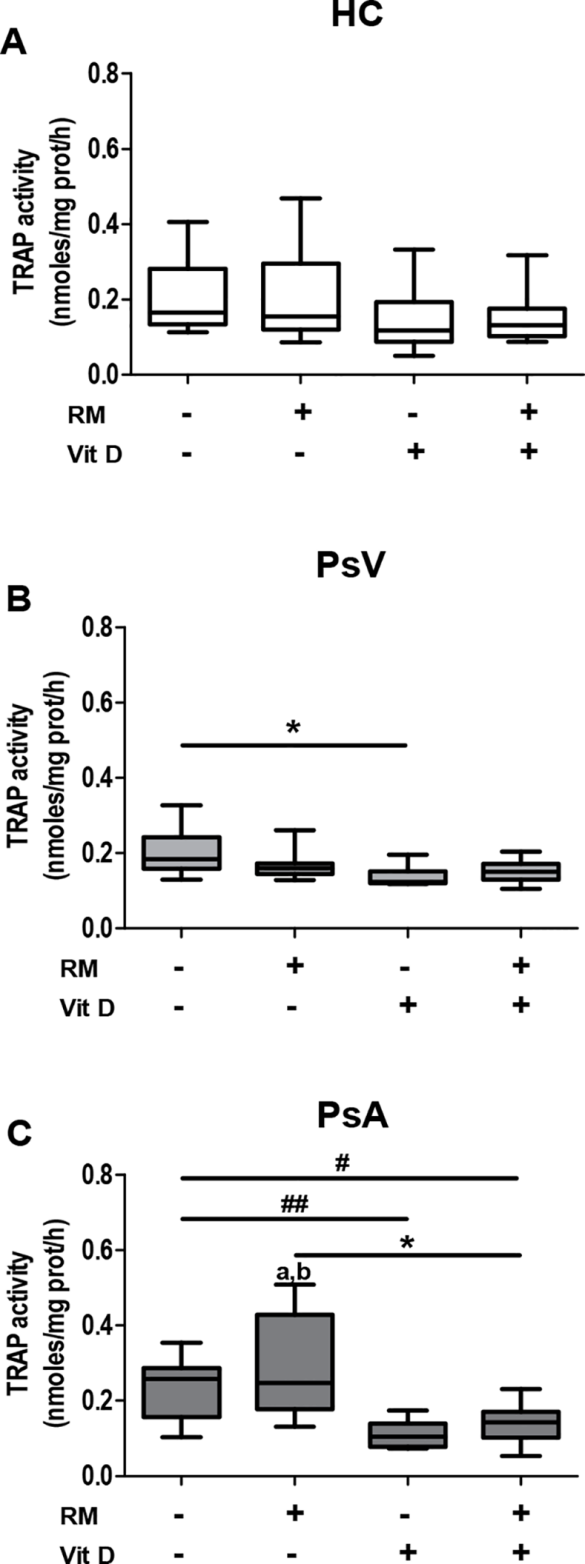
Effect of 1,25(OH)_2_D_3_ on TRAP activity from psoriasis and control PBMCs during osteoclastogenesis. PBMCs from A) healthy controls (HC, n = 14), and patients with B) psoriasis vulgaris (PsV, n = 9) and C) psoriatic arthritis (PsA, n = 11) were cultured with or without RANKL/M-CSF (RM) in the presence or in the absence of 1,25(OH)_2_D_3_ (Vit D); where n is the number of participants. TRAP enzymatic activity was measured in culture supernatants after 14 days. Box and whisker plots represent median with minimum to maximum values. *P<0.05 indicate statistically significant differences obtained by one-way ANOVA with Dunnetts’s multiple comparison test, and, #*P*<0.05 and ##*P*<0.01 only by Mann-Whitney or t-test. a*P*<0.05 indicate statistically significant differences between PsA and PsV cultured with RM obtained by one-way ANOVA with Dunnetts’s multiple comparison test, and, b*P*<0.05 between PsA and HC cultured with RM only by Mann-Whitney or t-test.

**Fig 4:**
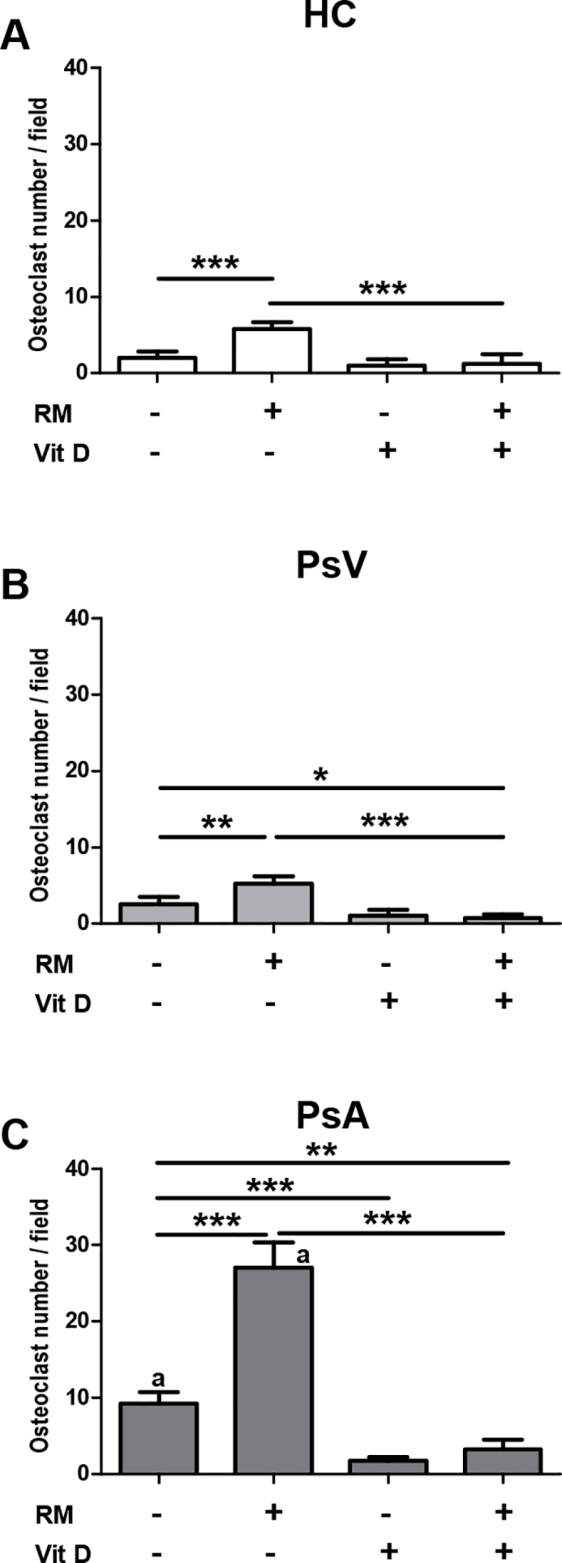
Effect of 1,25(OH)_2_D_3_ on TRAP+ osteoclast number from psoriasis and control PBMCs during osteoclastogenesis. PBMCs from A) healthy controls (HC, n = 4), and patients with B) psoriasis vulgaris (PsV, n = 4) and C) psoriatic arthritis (PsA, n = 4) were cultured with or without RANKL/M-CSF (RM) in the presence or in the absence of 1,25(OH)_2_D_3_ (Vit D) where n represents the number of samples. TRAP+ multinucleated cells (≥ 3 nuclei) per visual field, identified as mature osteoclasts, were counted after 14 days. Columns represent mean ± SD. *P<0.05, **P<0.01 and ***P<0.001 indicate statistically significant differences obtained by one-way ANOVA with Dunnetts’s multiple comparison test. a*P*<0.001 indicate statistically significant differences between PsA and PsV, and, PsA and HC cultured with and without RM, obtained by one-way ANOVA with Dunnetts’s multiple comparison test.

PBMCs from controls and patients with PsV and PsA were incubated with or without RANKL/M-CSF (RM) in the presence or absence of 1,25(OH)_2_D_3_ (Vit D). Thereafter, TRAP enzymatic activity was measured in order to assess osteoclast activity. No differences were observed between PBMCs from patients and controls under culture control conditions, but in the presence of RM, PBMCs of PsA showed higher TRAP enzymatic activity compared with PBMCs from PsV and controls (*P*<0.05, *P* = 0.041, respectively; Fig 3A–3C). However, a significant higher number of PsA multinucleated TRAP+ cells per field was observed in the absence or presence of RM compared with PBMCs of PsV and controls (*P*<0.001, Fig 4A–4C). Next the effect of 1,25(OH)_2_D_3_ on TRAP activity was analysed in PBMCs of HC, PsV and PsA. We observed no effect of 1,25(OH)_2_D_3_ in the case of control PBMCs while 1,25(OH)_2_D_3_ slightly inhibited TRAP activity from PBMCs of PsV compared with culture control conditions (*P*<0.05). Furthermore, 1,25(OH)_2_D_3_ diminished TRAP activity in PBMCs of PsA, even in the presence of RM compared with culture control conditions (*P* = 0.008, *P* = 0.040, respectively) and with RM alone (*P*<0.05) (Fig 3A–3C). However, 1,25(OH)_2_D_3_ diminished the number of multinucleated TRAP+ cells from all groups in the presence of RM, but in a greater proportion and even in the absence of RM in PBMCs of PsA (*P*<0.001, Fig 4A–4C).

### Differential cytokine secretion during osteoclastogenesis *in vitro*

**Fig 5:**
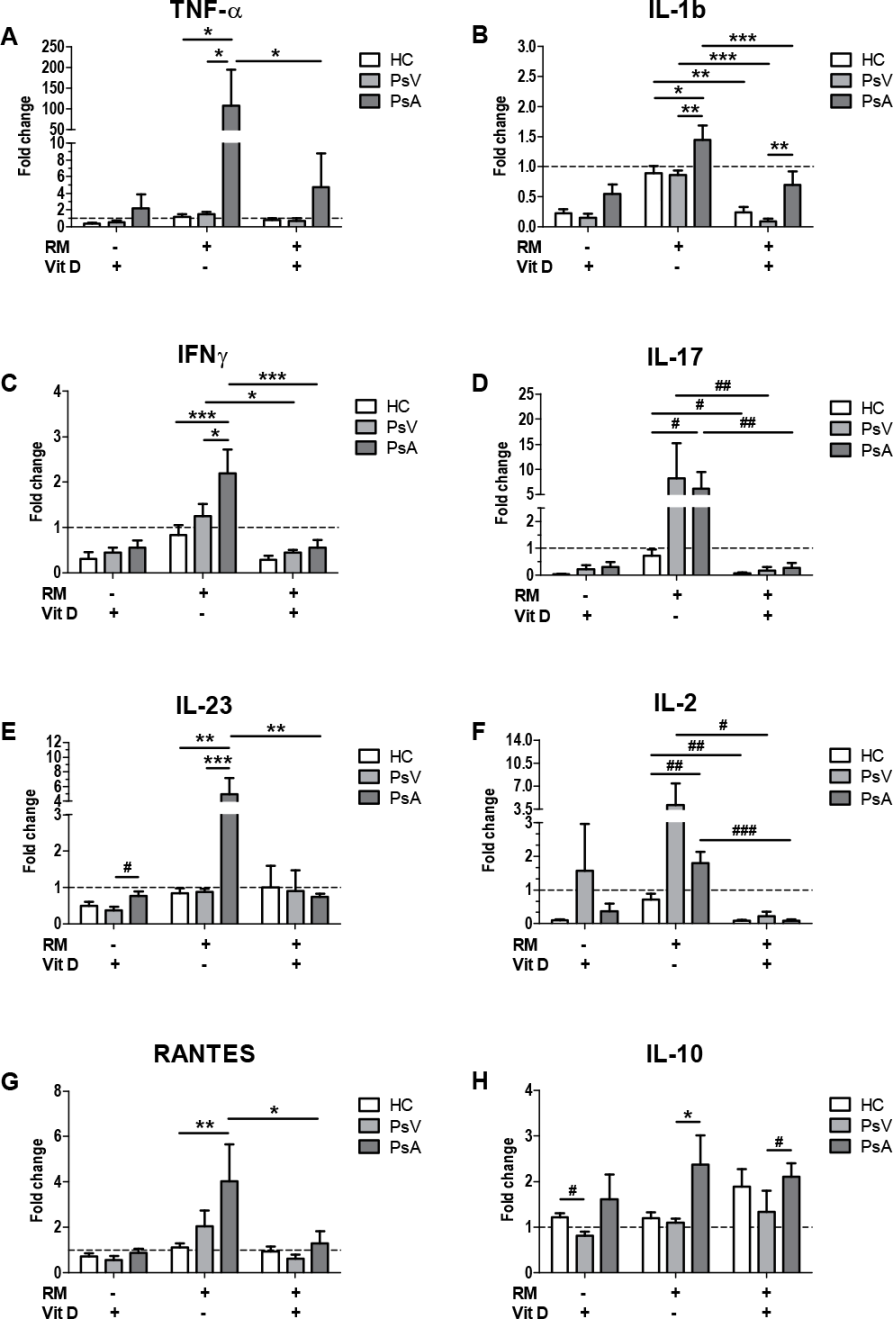
Effect of 1,25(OH)_2_D_3_ on cytokine secretion from PBMCs of psoriasis patients and controls. PBMCs from healthy controls (HC, white), and patients with psoriasis vulgaris (PsV, light grey) and psoriatic arthritis (PsA, dark grey) were cultured with or without RANKL/M-CSF (RM), and in the presence or in the absence of 1,25(OH)_2_D_3_ (Vit D). After 14 days supernatant concentrations of A) TNF-α, B) IL-1b, C) IFN-γ, D) IL-17, E) IL-23, F) IL-2, G) RANTES and H) IL-10 were measured by Q-Plex multiplex arrays. Columns represent mean ± SD of fold change of cytokine secretion compared to cytokine levels under culture conditions in the absence of RM and/or Vit D represented by dotted lines. HC, n = 5; PsV, n = 7; and PsA, n = 5; where n is the number of participants. Significant differences were obtained by two way ANOVA followed by Bonferroni post hoc test. *P<0.05, **P<0.01 and ***P<0.001 are statistical significant differences obtained by the Bonferroni post hoc test. #*P*<0.05, ##*P*<0.01 and ###*P*<0.001 indicate statistical significant differences only obtained by Mann-Whitney test.

Vitamin D plays an important role in the modulation of the immune system [19–24]. Therefore, we assessed the effect of vitamin D on cytokine secretion profile from psoriatic PBMCs upon osteoclastogenesis. Data was normalized due to baseline secretion differences between individual samples from the same group and treatment, as follows: For each sample, culture condition and cytokine, a fold change was calculated; equal to the cytokine concentration in the stimulated (RM, Vit D and RM+VitD) divided by the culture control condition (without RM, Vit D and RM+VitD) (Fig 5).

PBMCs of PsA in the presence of RM showed an increased secretion of cytokines such as TNF-α, IL-1b, IFN-γ, IL-17, IL-23, IL-2 and RANTES (91.83 fold, *P*<0.05; 1.62 fold, *P*<0.05; 2.64 fold, *P*<0.001; 8.59 fold, *P* = 0.032; 5.89 fold, *P*<0.01; 2.52 fold, *P* = 0.008; 3.63 fold, *P*<0.01, respectively) compared with PBMCs of HC (Fig 5A–5G). Furthermore, PBMCs of PsA showed higher secretion of TNF-α, IL-1b, IFN-γ, IL-23, IL-10 (71.49 fold, *P*<0.05; 1.69 fold, *P*<0.01; 1.76 fold, *P*<0.05; 5.68 fold, *P*<0.001; 2.16 fold, *P*<0.05, respectively) compared with PBMCs of PsV (Fig 5A–5C, 5E and 5H).

Addition of 1,25(OH)_2_D_3_ decreased levels of IL-1b (2.09 fold, *P*<0.0001; 9.85 fold, *P*<0.0001; 3.74 fold, *P*<0.01, respectively), IL-17 (22.77 fold, *P* = 0.008; 51.54 fold, *P* = 0.007; 11.93 fold, *P* = 0.02, respectively) and IL-2 (20.0 fold, *P*<0.0001; 18.51 fold, *P* = 0.035; 8.11 fold, *P* = 0.008, respectively) by PBMCs of PsA, PsV and HC; IFN-γ by PsA and PsV (3.87 fold, *P*<0.0001; 2.79 fold, *P*<0.05, respectively); and TNF-α, IL-23 and RANTES by PsA (22.83 fold, *P*<0.05; 6.69 fold, *P*<0.01; 3.12 fold, *P*<0.05, respectively) compared with RM alone (Fig 5A–5G). On the other hand, PBMCs of PsA secreted higher levels of IL-1b and IL-10 in the presence of RM+Vit D (7.95 fold, *P*<0.01 and 1.58 fold, *P* = 0.048, respectively) compared with PsV (Fig 5B and 5H). However, the effect of 1,25(OH)_2_D_3_ in the presence of RM showed no differences in any of the cytokines compared with the effect of 1,25(OH)_2_D_3_ alone (Fig 5).

## Discussion

For decades the beneficial effects of sunlight in the treatment of skin diseases such as psoriasis vulgaris, and immune system modulation are known. After sunlight exposure of the skin, 7-dehydrocholesterol converts to pre-vitamin D_3_ [33], which is hydroxylated in the liver into 25-hydroxyvitamin D_3_ [25-(OH)D_3_] [34] and in the kidney into 1,25-dihydroxyvitamin D_3_ [1,25(OH)_2_D_3_], the active vitamin D metabolite involved in extracellular calcium homeostasis and bone metabolism. Furthermore, lack of sun exposure and adequate vitamin D supply results in vitamin D deficiency and musculoskeletal pathologies [35,36]. Epidemiological studies show that vitamin D deficiency is frequent in patients with psoriasis vulgaris and psoriatic arthritis [37–40]. Although phototherapy and topical application of vitamin D analogs have been used as first-line treatment with satisfactory results in the treatment of psoriasis vulgaris [1,2], therapies including vitamin D in patients with psoriatic arthritis are currently not available. Pilot studies with oral vitamin D analogs supplementation have demonstrated efficacy and safety in the treatment of psoriatic arthritis [41,42] and psoriasis vulgaris [43,44]. However, there have been only few randomized controlled trials on vitamin D supplementation, which include patients with psoriasis vulgaris but none with psoriatic arthritis [45–48]. Moreover, the only finished randomized controlled trial did not show any vitamin D benefit [45] while the other three trials have not yet been completed [46–48]. More studies are needed to confirm if correction of deficiency would result in a statistically significant clinical improvement. On the other hand, to date, many studies have focused on the evaluation of the effects of cytokines and immune cells on osteoclast differentiation, but few on the reciprocal effects of osteoclasts on immune cells under physiological and pathological conditions. We aimed to study the osteoclastogenic potential and cytokine profile of PBMCs from patients with psoriasis vulgaris and psoriatic arthritis in response to 1,25(OH)_2_D_3_.

Osteoclasts are important players in calcium homeostasis, and calcitonin is a hormone which reduces blood calcium inhibiting bone resorption as well as osteoclast formation [12]. We assessed the calcium and bone resorption marker levels in patients with psoriasis vulgaris and psoriatic arthritis. No differences in serum levels of 1,25(OH)_2_D_3_, calcium and CT between patients and controls were observed. However, our results showed a strong direct positive Spearman’s correlation between CT and 1,25(OH)_2_D_3_ in HC, and a less strong Spearman’s coeficient of about 0.7 in all psoriasis patients.

CTSK, highly expressed in osteoclasts, is involved in the degradation of type I collagen [11], and OCN secreted by osteoblasts, is involved in bone formation [49]. Besides CTX-1/OCN ratios, we determined also the CTX-1/CTSK ratios in psoriasis patients and controls as CTX-1 is a product of collagen type I degradation by CTSK [32]. Such a ratio has not been reported before. We confirmed an altered bone remodelling in PsA patients demonstrated by higher CTX-1, lower OCN serum levels, and also higher CTX-1/OCN and CTX-1/CTSK ratios compared with controls. As CTX-1 is present in scar tissue as well as in dermis, tendons and ligaments, it was not surprising that also PsV patients with an impaired skin barrier and altered wound healing had slightly higher serum levels of CTX-1/OCN and CTX-1/CTSK ratios as controls. However, values were still lower compared with those found in patients with PsA. Although, recently CTSK has been found highly expressed in psoriasis patient skin and involved in development of psoriasis-like skin lesions, inflammation and bone erosion in mouse models [50,51], we found only significant higher CTSK levels in patients with PsA compared with PsV. Furthermore, our results showed a negative high coefficient correlation between CT and CTSK serum levels in patients with PsA but no correlation in case of PsV and HC. It is known that CT inhibits bone resorption in response to high serum calcium levels [52]. Our results suggest an altered osteoclast resorption response to hypercalcemic serum conditions which might explain partially an increase in the degradation of type 1 collagen by CTSK in the case of patients with PsA.

Psoriatic arthritis has been associated with high numbers of circulating osteoclast precursors; and osteoclasts, differentiated from those precursors have increased resorptive activity [29]. 1,25(OH)_2_D_3_ promotes osteoclast differentiation by increasing expression of mature osteoclast-associated genes [53] and stimulating bone resorption [54,55], but also it decreases resorptive activity in osteoclasts matured and maintained in the presence of 1,25(OH)_2_D_3_ [56]. Moreover, 1,25(OH)_2_D_3_ inhibits RANKL-induced osteoclastic differentiation in the absence of osteoblasts *in vitro* [57]. Our results showed high numbers per field of multinucleated TRAP+ cells in the presence of RM by PBMCs of PsA, as already reported [58]. In addition, we observed high TRAP activity in the presence of RM by PBMCs of PsA, and also high multinucleated TRAP+ cell numbers in the absence of RM. Moreover, to our knowledge, it has not been reported before that 1,25(OH)_2_D_3_ inhibited TRAP activity by PBMCs of PsV and PsA, and reduced TRAP+ cell numbers in a greater extent by PsA. No increases in TRAP activity in the presence of RM, but high multinucleated TRAP+ cell numbers by all PBMCs were observed. These discrepancies could be explained by the heterogeneity of the PBMCs population [59] with osteoclast precursors which in turn comprise several subpopulations, even with different proliferative capacities [60,61]. In addition, TRAP is expressed and secreted also by other cells than mature osteoclasts such as macrophages and dendritic cells [62].

Immune cells play an important role in the pathology of psoriasis, and together with osteoclasts in the pathology of joint inflammation in patients with PsA. Osteoclasts can present antigens to T cells [63], secrete chemokines such as IL-8 and RANTES [64] and recruit T cells *in vitro* [65]. Osteoclasts may express IL-6, TNF-α, IL-1α, IL-1β and macrophage inflammatory protein-1α *in vivo* [66–68]. Anti-CD3-stimulated PBMCs of PsA secreted higher IL-2 IFN-γ and IL-10, lower IL-17, and showed no differences in TNF-α secretion [69]. Our results showed that RM-stimulated PBMCs of PsA secreted higher levels of proinflammatory cytokines such as TNF-α, IL-1b, IFN-γ, IL-17, IL-23, IL-2 and RANTES compared with control PBMCs, and additionally higher TNF-α, IL-1b, IFN-γ, IL-23 and IL-10 compared with PBMCs of PsV, suggesting that these cytokines may contribute to the joint inflammation scenery in patients with PsA. No differences in any cytokine secretion were observed between RM-stimulated PBMCs of PsV and HC; which correspond to those authors who reported no differences in IL-10, IL-1β and IL-23 levels of LPS-stimulated PBMCs from cutaneous psoriasis [70]. Here we show that PBMCs from patients with PsV and PsA have differential cytokine secretion in response to 1,25(OH)_2_D_3_. In the presence of RM, 1,25(OH)_2_D_3_ decreased levels of IL-1b, IL-17 and IL-2 by PsA, PsV and HC; IFN-γ by PsA and PsV; and TNF-α, IL-23 and RANTES by PsA after 14 days compared with RM-stimulated PBMCs. However, 1,25(OH)_2_D_3_ inhibited secretion of any tested cytokine regardless of the presence or absence of RM.

Taken together, our study confirmed an altered bone remodelling in patients with PsA characterized by lower serum OCN, and higher CTSK, CTX-1, CTX-1/OCN and CTX-1/CTSK ratios compared with patients with PsV and controls. Additionally, patients with PsA have a negative correlation between CT and CTSK. PBMCs from patients with PsA have higher TRAP enzymatic activity in the presence of RM, but definitely showed increased sensitivity to the inhibition by 1,25(OH)_2_D_3_. Under RM stimulation, PBMCs of PsA produced higher levels of proinflammatory cytokines compared with PBMCs of HC, but also TNF-α, IL-1b, IFN-γ, IL-23 and the anti-inflammatory cytokine IL-10 compared with PBMCs of PsV; with differences in response to 1,25(OH)_2_D_3_.

Our data provides new insight into the different cytokine secretion profiles of PBMCs in the circulation of patients with PsA and PsV, and differences in their capacity to differentiate into osteoclasts and respond to 1,25(OH)_2_D_3_. Therefore, these data also suggest the development of therapeutic strategies including vitamin D for patients with psoriatic arthritis.

## Supporting Information

S1 Table Subjects demographics from patients with PsV and PsA, and healthy controls. Data from age variable are shown as mean ± SD. *One-way ANOVA with Bonferroni’s multiple comparison test. **Two-way ANOVA with Bonferroni post test analysis.
(DOC)
