# Supplementary material for: Effect of Vitamin D on Peripheral Blood Mononuclear Cells from Patients with Psoriasis Vulgaris and Psoriatic Arthritis
Source: PLoS One. 2016 Apr 6;11(4):e0153094. doi: 10.1371/journal.pone.0153094 (PMC4822855; doi:10.1371/journal.pone.0153094)
Supplement: S1 Table — Data from age variable are shown as mean ± SD. *One-way ANOVA with Bonferroni’s multiple comparison test. **Two-way ANOVA with Bonferroni post test analysis. (DOC) [file pone.0153094.s001.doc]

| **S1 Table. Subjects demographics from patients with PsV and PsA, and** | | | | |
| --- | --- | --- | --- | --- |
| **healthy controls.** | |  |  |  |
| **Demographics** | **HC (n=15)** | **PsV (n=9)** | **PsA (n=12)** | ***P*** |
| **Age (years)** | 42.27 ± 9.48 | 50.22 ± 17.19 | 51.08 ± 10.96 | 0.139 ^a, b, c^ * |
| **Male age (years)** | 40.86 ± 9.51 | 42.33 ± 14.28 | 49.57 ± 9.03 | <0.05 **,# |
| **(%)** | (46.67) | (66.67) | (58.33) | >0.05 ^a, b, c^ |
| **Female age (years)** | 43.50 ± 9.91 | 66.00 ± 10.58 | 53.20 ± 14.08 | <0.05 **,# |
| **(%)** | (53.33) | (33.33) | (41.67) | <0.05 ^a^ |
|  |  |  |  | >0.05 ^b, c^ |
| HC, healthy controls; PsV, psoriasis vulgaris; PsA, psoriatic arthritis; Data for age variable are | | | | |
| shown as mean ± SD. *One-way ANOVA with *post hoc* test. **Two-way ANOVA with *post hoc* | | | | |
| test. # no interaction between age and sex. Bonferroni post test analysis: ^a^ PsV vs. HC, ^b^ PsA | | | | |
| vs. HC, ^c^ PsA vs. PsV. | |  |  |  |
